# Epidemiology of cruciate ligament surgery in Japan: A repeated cross-sectional study from 2014 to 2021

**DOI:** 10.1371/journal.pone.0288854

**Published:** 2023-12-22

**Authors:** Shota Uchino, Masataka Taguri

**Affiliations:** 1 Department of Data Science, Graduate School of Data Science, Yokohama City University, Kanazawa-ku, Yokohama, Japan; 2 REHASAKU Co., Ltd., Minato-ku, Tokyo, Japan; 3 Department of Health Data Science, Tokyo Medical University, Shinjuku-ku, Tokyo, Japan; Kyushu University, JAPAN

## Abstract

Understanding the incidence and trends of cruciate ligament (CL) surgeries in Japan is crucial for providing effective healthcare services. This study aimed to use open data available from the National Database of Health Insurance Claims and Specific Health Checkups of Japan (NDB) to analyze changes in CL surgeries over time and the characteristics of the Japanese population by sex and age. We retrospectively identified CL surgeries of the knee joint registered from April 2014 to March 2022 using the NDB open data. Data on sex, age, and practice were extracted to determine the number of cases per 100,000 population. Trends in the annual incidence of CL surgeries were evaluated using Poisson regression analysis. A total of 142,931 CL surgeries were performed from 2014 to 2021, with arthroscopic ligament reconstruction accounting for 98% of cases. The number of surgeries significantly increased from 16,975 in 2014 to 19,735 in 2019 (P<0.001). CL surgeries were most common in the 15–19 and 20–29 years age groups, with variations between males and females. The incidence of CL surgery in Japan has increased, with characteristics varying by sex and age, including middle-aged and older patients. Further investigation of general patterns in CL surgery in Japan would be valuable.

## Introduction

The availability of medical and national big data has enabled the determination of ligament injury and surgery incidence [1–9]. Recent trends show a decline in knee ligament surgeries in Italy over the past 4 years, with higher occurrence among males [1, 2]. In contrast, South Korea has seen an upward trend in surgical cases, particularly among females [3, 4]. Both countries observed the highest number of surgeries in patients in their 20s [1–4]. These studies provide epidemiological models for the generalization of disease incidence and help improve treatment strategies and healthcare services to enhance the overall quality of life for patients. However, these are differences in incidence rates and sex- and age-specific trends between countries, emphasizing the need for comprehensive investigations within the Japanese population.

In Japan, an epidemiological study using insurance records of middle and high school athletes reported 30,458 anterior cruciate ligament (ACL) injuries (0.81 per 1000 athlete-years) over a 10-year period from 2005 to 2014. The incidence rate of ACL injuries was reported to be 2.8 times higher in female athletes than in male athletes [10]. This previous study focused on populations at high risk for ACL injury, leaving the incidence and post-injury response of other populations unknown.

To understand the incidence and trends in Japan, this study surveyed the population without age or other restrictions. Recent epidemiological studies have utilized the National Database of Health Insurance Claims and Specific Health Checkups of Japan (NDB), established by the Ministry of Health, Labour and Welfare (MHLW), and the NDB open data, which is a more generalized compilation of the NDB, have been actively conducted in the field of locomotor system [11–13]. Analyzing longitudinal trends in specific diseases from this big data helps specialists gain a deeper understanding of injuries. This data provides valuable insights for prevention strategies and treatment in the field of cruciate ligament (CL) surgeries.

The purpose of this study was to examine annual trends in the number, sex, and age distributions of CL surgeries using NDB open data from 2014 to 2021. In formulating our hypotheses, we referred to trends observed in neighboring countries [1–4]. In particular, the study by Chung et al., using data from the Health Insurance Review and Assessment Service (HIRA) in Korea, highlights a marked increase in CL surgeries over the past few years. Based on this evidence, we hypothesized that CL surgery will show a similar increasing trend in Japan, especially among women and young adults, consistent with the patterns observed in neighboring regions.

## Materials and methods

This population-based repeated cross-sectional study used the NDB open data provided by the MHLW from April 2014 to March 2022.

Data on the "number of calculations by sex and age group" for "operation (Code K)" [14–21] were obtained from the NDB open data website. Four categories of knee joint CL surgeries were identified: ligament tear suture (K074), arthroscopic ligament tear suture (K074-2), ligament reconstruction (K079), and arthroscopic ligament reconstruction (K079-2). The number of registered CL surgeries was analyzed by fiscal year, sex, and age (5-year interval). No detailed analyses were performed if the number of registered CL surgeries in <10 cases had no specific values shown. Data on sex and age were calculated as the number of surgeries per 100,000 population using population estimates from the Statistics Bureau of the Ministry of Internal Affairs and Communications based on population data as of October 1 of each year [22].

Changes in the number of surgeries on the CL of the knee joint over time were assessed using Poisson regression, with the number of surgeries as the outcome variable, and the year of surgery as the explanatory variable. The supporting information file includes the STROBE checklist details (S1 Table). The NDB open data is a freely available tabulation table created by the MHLW based on the actual status of medical care in Japan and the results of specific health checkups. Data are compiled and published based on existing anonymized NDB, and since there is no corresponding table, individuals cannot be identified. Thus, ethical approval was unnecessary, and obtaining informed consent was not required for this study.

## Results

**Fig 1:**
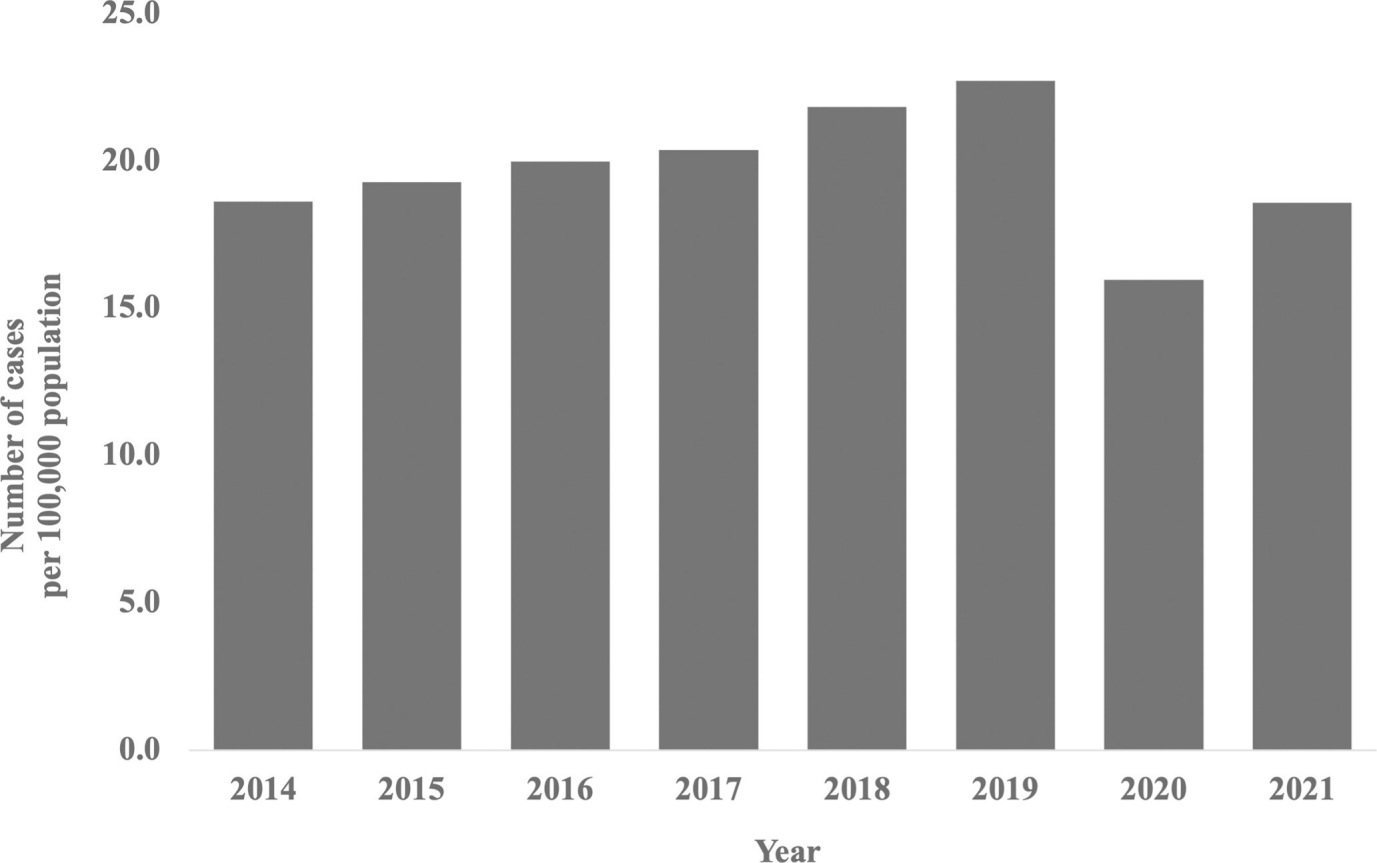
Number of registered arthroscopic ligament reconstruction procedures per 100,000 population from 2014 to 2021.

In total, 142,931 CL surgeries were performed from April 2014 to March 2022, with 139,596 (98%) being arthroscopic ligament reconstruction. The remaining procedures included ligament reconstruction and arthroscopic ligament tear sutures, each accounting for approximately 1.0% of the total number of surgeries performed, with very few cases of ligament tear sutures (Table 1). The number of arthroscopic ligament reconstruction increased significantly from 16,997 (18.3 cases/100,000 population) in 2014 to 19,774 (22.1 cases/100,000 population) in 2019, surpassing the number of all other techniques (P<0.001). However, there was a sharp decline from 2019 to 2021 (Fig 1).

**Table 1:** Annual number of cruciate ligament surgeries in the NDB open data from 2014 to 2021.

Category, year	2014	2015	2016	2017	2018	2019	2020	2021
**Ligament tear suture**	26	24	15	20	16	19	14	18
**Arthroscopic ligament tear suture**	225	204	228	267	213	227	173	177
**Ligament reconstruction**	289	229	230	209	169	151	101	91
**Arthroscopic ligament reconstruction**	16,997	17,507	18,129	18,241	19,277	19,774	13,793	15,878
**Total**	17,537	17,964	18,602	18,737	19,675	20,171	14,081	16,164

Numbers less than 10 are not displayed in the NDB to protect personal information (S2–S9 Tables). Therefore, only arthroscopic ligament reconstruction procedures, for which detailed data were available during the survey period, were included in the analysis by sex and age (S5 and S9 Tables).

**Fig 2:**
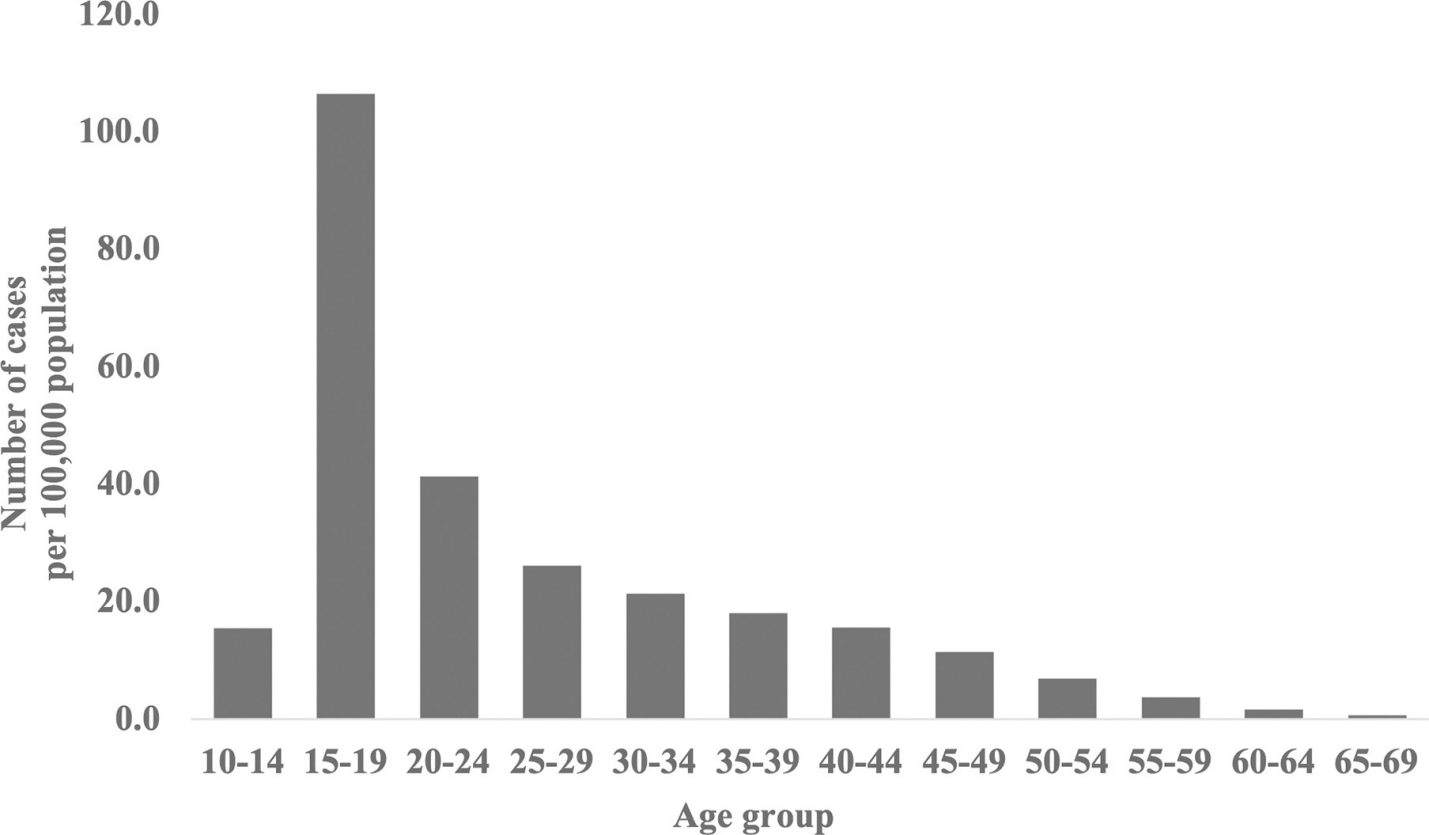
Number of registered arthroscopic ligament reconstruction procedures per 100,000 population by age group from 2014 to 2021.

Due to low registration numbers, data for the 0–9 and >70 years age groups were incomplete. For the 10–69 years age group, the highest number of registrations was observed in the 15–19 years age group (104.6 cases/100,000 population), followed by the 20–24 years age group (39.4 cases/100,000 population). We noted that registrations declined with age (Fig 2).

**Fig 3:**
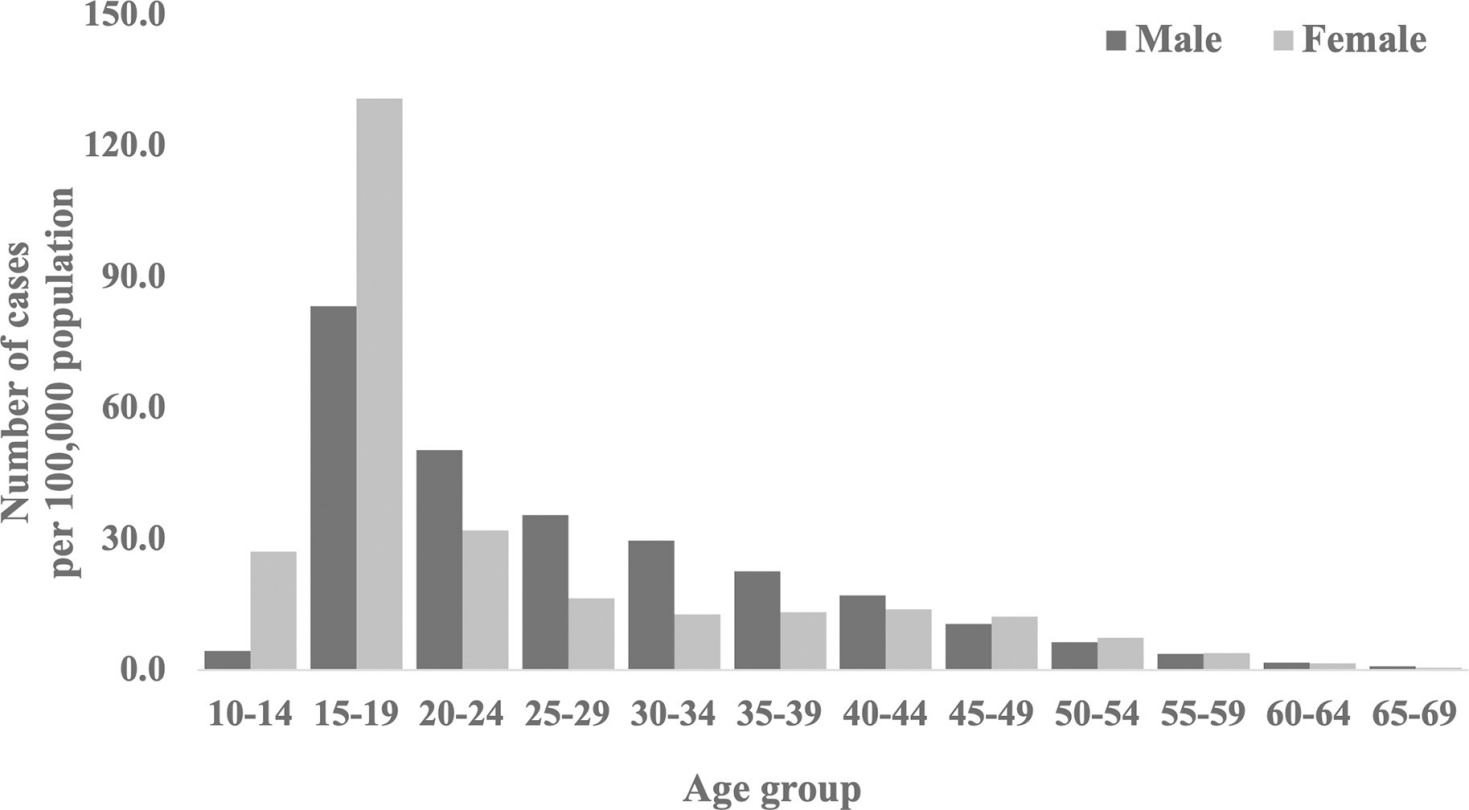
Number of registered arthroscopic ligament reconstruction procedures per 100,000 population.

Regarding sex, females outnumbered males by a factor of 5.9 in the 10–14 years age group and by 1.5 in the 15–19 years age group. In contrast, males were 1.7 times more likely than females to be enrolled in the 20–24 years age group and more than twice as likely as females to be enrolled in the 25–29 and 30–34 years age groups. Registration numbers were similar for other age groups (Fig 3).

## Discussion

This study aimed to 1) use sex and age-specific data on CL surgery to reveal annual trends and 2) identify differences in the incidence of CL surgery by sex and age in Japan. The NDB open data employed in this study is accessible to anyone through the MHLW website [14–21]. Going beyond a mere explanation of the data, this study adopted a data aggregation methodology that combines the demographic data in Japan, enabling time-series analysis unaffected by population fluctuations. Consequently, it allows for comparisons across different regions, providing unprecedented data on CL surgeries in Japan. Given the limited epidemiological studies in this field in Japan, the ability to compare across regions and present population-based data in Japan is valuable. Moreover, by leveraging Open Data to shape research outcomes, we emphasize our commitment to observational studies supported by comprehensive databases. As we hypothesized, we observed an increasing trend in the number of surgeries from 2014 to 2019, but a decrease was observed in 2020. This could be due to the impact of COVID-19. The global pandemic has imposed restrictions on various aspects of daily life, including engagement in sports activities. Furthermore, several factors have been suggested, including concerns about in-person consultations, decreased patient visits to orthopedic and sports medicine clinics, and a shift to telemedicine [23, 24]. Despite the influence of this social context, it is noteworthy that most CL surgeries were arthroscopic ligament reconstruction procedures, which constitutes a significant finding of this study. With the advancement of medical technology, arthroscopic surgery, which enables a detailed examination of the interior of a joint during surgery, has become increasingly prevalent [25, 26]. This development may have led to a rise in the number of arthroscopic procedures in Japan.

Arthroscopic ligament reconstruction was found to vary by sex and age. Overall, the incidence was higher among males. Conversely, females often undergo surgery during their teenage years, potentially due to the disparity in growth rates between males and females. Height growth in Japanese females reaches its peak around 2 years earlier than in males [27]. Variations in growth rates may influence the timing of surgery. However, it should be noted that CL surgery has been the subject of intense debate regarding its potential impact on growth retardation [28, 29]. Other factors, such as the inability of females to be active in sports at times due to childbirth, may be related to the sex difference. Another finding was that the incidence of surgery among middle-aged and older adults had also increased. Injuries resulting from sports activities, everyday sprains, and falls are common among middle-aged and older adults [30]. This can be attributed to increased health awareness and higher physical activity levels. Additionally, there is a higher risk of knee joint dysfunction and secondary injuries (such as meniscus and cartilage damage) [31, 32]. Good outcomes of CL surgery in middle-aged and older patients have also been reported [33, 34]. Consequently, many patients may choose surgery to regain their previous level of activity. In Japan, like in other countries, the indications for surgery have likely expanded due to changes in individual lifestyles and advancements in medical technology.

This study has several limitations. First, it failed to provide details on the number of ACL and Posterior cruciate ligament (PCL) surgeries as a categorization of the CL surgeries. Second, tissue injuries other than those caused by CL surgery were not identified, as they cannot be used for ligament and meniscus surgeries for health claims. Third, it was impossible to analyze information other than medical practice, sex, and age. Finally, it should be noted that this dataset does not include information on social welfare for cases related to traffic accidents, occupational injuries, or medical assistance; these elements are not part of the current data.

In future studies, we aim to investigate the number of injuries and reconstruction surgeries separately for ACL and PCL injuries. We would also like to clarify the characteristics of Japanese patients through comparison with overseas reports. We believe organizing information on knee joint ligament injuries will lead to novel suggestions for rehabilitation and prevention programs. This initiative utilizes an Open Database, and we strongly believe that this approach is applicable to other conditions, contributing to the advancement of epidemiological research.

## Conclusion

There is an increased incidence of CL surgery in Japan, along with sex and age characteristics. This trend is not limited to younger age groups but is also observed in middle-aged and older age groups. In the future, more detailed data should be extracted from medical databases to identify insights for optimal treatment strategies for specific populations.

## Supporting information

S1 Table STROBE statement—a checklist of items that should be included in reports of observational studies. (DOCX)

S2 Table Annual registrations of ligament tear suture (K074) according to age groups from 2014 to 2021. To avoid the identification of individuals, aggregate units that are <10 in principle are not included.
(DOCX)

S3 Table Annual registrations of arthroscopic ligament tear suture (K074-2) according to age groups from 2014 to 2021. To avoid the identification of individuals, aggregate units that are <10 in principle are not included.
(DOCX)

S4 Table Annual registrations of ligament reconstruction (K079) according to age groups from 2014 to 2021. To avoid the identification of individuals, aggregate units that are <10 in principle are not included.
(DOCX)

S5 Table Annual registrations of arthroscopic ligament reconstruction (K079-2) according to age groups from 2014 to 2021. To avoid the identification of individuals, aggregate units that are <10 in principle are not included.
(DOCX)

S6 Table Annual registrations of ligament tear suture (K074) per 100,000 population according to sex and age groups from 2014 to 2021. To avoid the identification of individuals, aggregate units that are <10 in principle are not included.
(DOCX)

S7 Table Annual registrations of arthroscopic ligament tear sutur (K074-2) per 100,000 population according to age groups from 2014 to 2021. To avoid the identification of individuals, aggregate units that are <10 in principle are not included.
(DOCX)

S8 Table Annual registrations of ligament reconstruction (K079) per 100,000 population according to age groups from 2014 to 2021. To avoid the identification of individuals, aggregate units that are <10 in principle are not included.
(DOCX)

S9 Table Annual registrations of arthroscopic ligament reconstruction (K079-2) per 100,000 population according to age groups from 2014 to 2021. To avoid the identification of individuals, aggregate units that are <10 in principle are not included.
(DOCX)
