# Supplementary material for: Epidemiology of cruciate ligament surgery in Japan: A repeated cross-sectional study from 2014 to 2021
Source: PLoS One. 2023 Dec 22;18(12):e0288854. doi: 10.1371/journal.pone.0288854 (PMC10745212; doi:10.1371/journal.pone.0288854)
Supplement: S1 Table — (DOCX) [file pone.0288854.s001.docx]

**S1 Table. STROBE Statement—checklist of items that should be included in reports of observational studies**

|  | Item No. | Recommendation | Page  No. | Relevant text from manuscript |
| --- | --- | --- | --- | --- |
| **Title and abstract** | 1 | (*a*) Indicate the study’s design with a commonly used term in the title or the abstract | 1 | A repeated cross-sectional study |
|  |  | (*b*) Provide in the abstract an informative and balanced summary of what was done and what was found | 2 | Abstract |
| Introduction | | | |  |
| Background/rationale | 2 | Explain the scientific background and rationale for the investigation being reported | 3 | Introduction Lines 34−47 |
| Objectives | 3 | State specific objectives, including any prespecified hypotheses | 4 | Introduction Lines 56−62 |
| Methods | | | |  |
| Study design | 4 | Present key elements of study design early in the paper | 5 | Methods Lines 64−65 |
| Setting | 5 | Describe the setting, locations, and relevant dates, including periods of recruitment, exposure, follow-up, and data collection | 5 | Methods Lines 64−65 |
| Participants | 6 | (*a*) *Cohort study*—Give the eligibility criteria, and the sources and methods of selection of participants. Describe methods of follow-up  *Case-control study*—Give the eligibility criteria, and the sources and methods of case ascertainment and control selection. Give the rationale for the choice of cases and controls  *Cross-sectional study*—Give the eligibility criteria, and the sources and methods of selection of participants | 5 | Methods Lines 66−70 |
|  |  | (*b*) *Cohort study*—For matched studies, give matching criteria and number of exposed and unexposed  *Case-control study*—For matched studies, give matching criteria and the number of controls per case | N/A (Not a matched study) |  |
| Variables | 7 | Clearly define all outcomes, exposures, predictors, potential confounders, and effect modifiers. Give diagnostic criteria, if applicable | 5 | Methods Lines 69−70 |
| Data sources/ measurement | 8* | For each variable of interest, give sources of data and details of methods of assessment (measurement). Describe comparability of assessment methods if there is more than one group | 5 | Methods Lines 66−77 |
| Bias | 9 | Describe any efforts to address potential sources of bias | N/A |  |
| Study size | 10 | Explain how the study size was arrived at | N/A (We took all eligible population) |  |

Continued on next page

| Quantitative variables | 11 | Explain how quantitative variables were handled in the analyses. If applicable, describe which groupings were chosen and why | 5 | Methods Lines 69−74 |
| --- | --- | --- | --- | --- |
| Statistical methods | 12 | (*a*) Describe all statistical methods, including those used to control for confounding | 5 | Methods Lines 75−77 |
|  |  | (*b*) Describe any methods used to examine subgroups and interactions | N/A (Not done) |  |
|  |  | (*c*) Explain how missing data were addressed | N/A (Not done) |  |
|  |  | (*d*) *Cohort study*—If applicable, explain how loss to follow-up was addressed  *Case-control study*—If applicable, explain how matching of cases and controls was addressed  *Cross-sectional study*—If applicable, describe analytical methods taking account of sampling strategy | 5 | Methods Lines 75−77 |
|  |  | (*e*) Describe any sensitivity analyses | N/A (Not done) |  |
| Results | | | | |
| Participants | 13* | (a) Report numbers of individuals at each stage of study—eg numbers potentially eligible, examined for eligibility, confirmed eligible, included in the study, completing follow-up, and analysed | 7 | Results Lines 84−87  Table 1 |
|  |  | (b) Give reasons for non-participation at each stage | N/A (This study is exhaustive survey) |  |
|  |  | (c) Consider use of a flow diagram | N/A (Not done) |  |
| Descriptive data | 14* | (a) Give characteristics of study participants (eg demographic, clinical, social) and information on exposures and potential confounders | 8 | Results Lines 87−108  Fig 2 and 3  S2−9 Tables |
|  |  | (b) Indicate number of participants with missing data for each variable of interest | N/A (This study is exhaustive survey) |  |
|  |  | (c) *Cohort study*—Summarise follow-up time (eg, average and total amount) | N/A (This is not a Cohort study) |  |
| Outcome data | 15* | *Cohort study*—Report numbers of outcome events or summary measures over time | N/A (This is not a Cohort study) |  |
|  |  | *Case-control study—*Report numbers in each exposure category, or summary measures of exposure | N/A (This is not a Case-control study) |  |
|  |  | *Cross-sectional study—*Report numbers of outcome events or summary measures | 7-8 | Results Lines 87−108  Fig 2 and 3  S2−9 Tables |
| Main results | 16 | (*a*) Give unadjusted estimates and, if applicable, confounder-adjusted estimates and their precision (eg, 95% confidence interval). Make clear which confounders were adjusted for and why they were included | N/A (Not done) |  |
|  |  | (*b*) Report category boundaries when continuous variables were categorized | 7-8 | Results Lines 87−108 |
|  |  | (*c*) If relevant, consider translating estimates of relative risk into absolute risk for a meaningful time period | N/A (Not done) |  |

Continued on next page

| Other analyses | 17 | Report other analyses done—eg analyses of subgroups and interactions, and sensitivity analyses | N/A (Not done) |  |
| --- | --- | --- | --- | --- |
| Discussion | | | | |
| Key results | 18 | Summarise key results with reference to study objectives | 10 | Discussion Lines 117−128 |
| Limitations | 19 | Discuss limitations of the study, taking into account sources of potential bias or imprecision. Discuss both direction and magnitude of any potential bias | 11 | Discussion Lines 152−158 |
| Interpretation | 20 | Give a cautious overall interpretation of results considering objectives, limitations, multiplicity of analyses, results from similar studies, and other relevant evidence | 13 | Conclusion Lines 167−170 |
| Generalisability | 21 | Discuss the generalisability (external validity) of the study results | N/A (Not done) |  |
| Other information | |  | | |
| Funding | 22 | Give the source of funding and the role of the funders for the present study and, if applicable, for the original study on which the present article is based | This work was supported by JSPS KAKENHI Grant Numbers JP21K02905. |  |

*Give information separately for cases and controls in case-control studies and, if applicable, for exposed and unexposed groups in cohort and cross-sectional studies.

**Note:** An Explanation and Elaboration article discusses each checklist item and gives methodological background and published examples of transparent reporting. The STROBE checklist is best used in conjunction with this article (freely available on the Web sites of PLoS Medicine at http://www.plosmedicine.org/, Annals of Internal Medicine at http://www.annals.org/, and Epidemiology at http://www.epidem.com/). Information on the STROBE Initiative is available at www.strobe-statement.org.
