# Supplementary material for: Epidemiology of cruciate ligament surgery in Japan: A repeated cross-sectional study from 2014 to 2021
Source: PLoS One. 2023 Dec 22;18(12):e0288854. doi: 10.1371/journal.pone.0288854 (PMC10745212; doi:10.1371/journal.pone.0288854)
Supplement: S3 Table — To avoid the identification of individuals, aggregate units that are <10 in principle are not included. (DOCX) [file pone.0288854.s003.docx]

**S3 Table. Annual registrations of arthroscopic ligament tear suture (K074-2) according to age groups from 2014 to 2021.**

| Year | Total | Age groups (Upper: Male, Lower: Female) | | | | | | | | | | | | | | | | | | |
| --- | --- | --- | --- | --- | --- | --- | --- | --- | --- | --- | --- | --- | --- | --- | --- | --- | --- | --- | --- | --- |
|  |  | 0−4 | 5−9 | 10−14 | 15−19 | 20−24 | 25−29 | 30−34 | 35−39 | 40−44 | 45−49 | 50−54 | 55−59 | 60−64 | 65−69 | 70−74 | 75−79 | 80−84 | 85−89 | ≥90 |
| 2014 | 225 | −　　− | −　　− | −　　10 | 26　　35 | 26　　10 | 18　　− | 10 　11 | 14　　− | −　　− | −　　− | −　　− | −　　− | −　　− | −　　− | −　　− | −　　− | −　　− | −　　− | −　　− |
| 2015 | 204 | −　　− | −　　− | −　　10 | 28　　32 | 11　　− | 10　　− | 16　　− | 10　　− | −　　12 | 10　　− | −　　− | −　　− | −　　− | −　　− | −　　− | −　　− | −　　− | −　　− | −　　− |
| 2016 | 228 | −　　− | −　　− | −　　− | 28　　40 | 27　　13 | 18　　− | 18　　− | −　　− | 10　　13 | −　　− | −　　− | −　　− | −　　− | −　　− | −　　− | −　　− | −　　− | −　　− | −　　− |
| 2017 | 267 | −　　− | −　　− | 15　　12 | 37　　40 | 23　　− | 12　　− | 12　　− | 23　　− | 14　　13 | −　　11 | −　　− | −　　− | −　　− | −　　− | −　　− | −　　− | −　　− | −　　− | −　　− |
| 2018 | 213 | −　　− | −　　− | −　　− | 26　　30 | 18　　11 | 19　　− | −　　− | 16　　− | 12　　− | −　　10 | −　　− | −　　− | −　　− | −　　− | −　　− | −　　− | −　　− | −　　− | −　　− |
| 2019 | 227 | −　　− | −　　− | 10　　− | 25　　40 | 19　　10 | 20 　− | 13　　− | −　　− | −　　− | −　　11 | −　　− | −　　− | −　　− | −　　− | −　　− | −　　− | −　　− | −　　− | −　　− |
| 2020 | 173 | −　　− | −　　− | −　　− | 17　　26 | 20　　− | 16　　− | 12　　− | 10　　− | −　　− | −　　− | −　　− | −　　− | −　　− | −　　− | −　　− | −　　− | −　　− | −　　− | −　　− |
| 2021 | 177 | −　　− | −　　− | −　　− | 23　　33 | 17　　− | 16　　10 | 13　　− | −　　− | −　　− | −　　− | −　　− | −　　− | −　　− | −　　− | −　　− | −　　− | −　　− | −　　− | −　　− |
