# Supplementary material for: Epidemiology of cruciate ligament surgery in Japan: A repeated cross-sectional study from 2014 to 2021
Source: PLoS One. 2023 Dec 22;18(12):e0288854. doi: 10.1371/journal.pone.0288854 (PMC10745212; doi:10.1371/journal.pone.0288854)
Supplement: S4 Table — To avoid the identification of individuals, aggregate units that are <10 in principle are not included. (DOCX) [file pone.0288854.s004.docx]

**S4 Table. Annual registrations of ligament reconstruction (K079) according to age groups from 2014 to 2021.**

| Year | Total | Age groups (Upper: Male, Lower: Female) | | | | | | | | | | | | | | | | | | |
| --- | --- | --- | --- | --- | --- | --- | --- | --- | --- | --- | --- | --- | --- | --- | --- | --- | --- | --- | --- | --- |
|  |  | 0−4 | 5−9 | 10−14 | 15−19 | 20−24 | 25−29 | 30−34 | 35−39 | 40−44 | 45−49 | 50−54 | 55−59 | 60−64 | 65−69 | 70−74 | 75−79 | 80−84 | 85−89 | ≥90 |
| 2014 | 289 | −　　− | −　　− | −　　11 | 39　　50 | 24　　14 | 16　　− | 24 − | 19　　12 | 14　　− | 12　　− | −　　− | −　　− | −　　− | −　　− | −　　− | −　　− | −　　− | −　　− | −　　− |
| 2015 | 229 | −　　− | −　　− | −　　− | 36　　38 | 25　　10 | 22　　− | 19　　− | 15　　− | 11　　13 | −　　− | −　　− | −　　− | −　　− | −　　− | −　　− | −　　− | −　　− | −　　− | −　　− |
| 2016 | 230 | −　　− | −　　− | −　　− | 49　　28 | 20　　13 | 16　　− | 15　　− | 13　　− | 10　　− | 10　　− | −　　− | −　　− | −　　− | −　　− | −　　− | −　　− | −　　− | −　　− | −　　− |
| 2017 | 209 | −　　− | −　　− | −　　− | 24　　43 | 18　　11 | 12　　− | −　　− | 11　　13 | 11　　− | −　　− | −　　− | −　　− | −　　− | −　　− | −　　− | −　　− | −　　− | −　　− | −　　− |
| 2018 | 169 | −　　− | −　　− | −　　− | 19　　35 | 18　　− | −　　− | −　　− | −　　− | −　　− | −　　− | −　　− | −　　− | −　　− | −　　− | −　　− | −　　− | −　　− | −　　− | −　　− |
| 2019 | 151 | −　　− | −　　− | −　　− | 24　　26 | −　　− | 10 　− | 10　　10 | −　　− | −　　− | −　　− | −　　− | −　　− | −　　− | −　　− | −　　− | −　　− | −　　− | −　　− | −　　− |
| 2020 | 101 | −　　− | −　　− | −　　− | 15　　18 | −　　− | −　　− | −　　− | −　　− | −　　− | −　　− | −　　− | −　　− | −　　− | −　　− | −　　− | −　　− | −　　− | −　　− | −　　− |
| 2021 | 91 | −　　− | −　　− | −　　− | 12　　18 | −　　− | −　　− | −　　− | −　　− | −　　− | −　　− | −　　− | −　　− | −　　− | −　　− | −　　− | −　　− | −　　− | −　　− | −　　− |
