# Supplementary material for: Epidemiology of cruciate ligament surgery in Japan: A repeated cross-sectional study from 2014 to 2021
Source: PLoS One. 2023 Dec 22;18(12):e0288854. doi: 10.1371/journal.pone.0288854 (PMC10745212; doi:10.1371/journal.pone.0288854)
Supplement: S5 Table — To avoid the identification of individuals, aggregate units that are <10 in principle are not included. (DOCX) [file pone.0288854.s005.docx]

**S5 Table. Annual registrations of arthroscopic ligament reconstruction (K079-2) according to age groups from 2014 to 2021.**

| Year | Total | Age groups (Upper: Male, Lower: Female) | | | | | | | | | | | | | | | | | | |
| --- | --- | --- | --- | --- | --- | --- | --- | --- | --- | --- | --- | --- | --- | --- | --- | --- | --- | --- | --- | --- |
|  |  | 0−4 | 5−9 | 10−14 | 15−19 | 20−24 | 25−29 | 30−34 | 35−39 | 40−44 | 45−49 | 50−54 | 55−59 | 60−64 | 65−69 | 70−74 | 75−79 | 80−84 | 85−89 | ≥90 |
| 2014 | 16,997 | −　　− | −　　− | 98 628 | 2,420 3,465 | 1,594 878 | 1,272　465 | 1,108　407 | 966　571 | 719　645 | 390　548 | 193　245 | 111　102 | 52　50 | 32　16 | −　　− | −　　− | −　　− | −　　− | −　　− |
| 2015 | 17,507 | −　　− | −　　− | 101 734 | 2,408 3,772 | 1,571　914 | 1,224　503 | 1,124　425 | 922　526 | 782　691 | 402　506 | 252　242 | 119　98 | 57　53 | 37　24 | −　　− | −　　− | −　　− | −　　− | −　　− |
| 2016 | 18,129 | −　　− | −　　− | 118 792 | 2,543 4,071 | 1,615　964 | 1,146　534 | 1,054　449 | 929　541 | 784　653 | 425　559 | 229　260 | 138　147 | 50　48 | 37　24 | −　　− | −　　− | −　　− | −　　− | −　　− |
| 2017 | 18,241 | −　　− | −　　− | 128 763 | 2,492 3,977 | 1,562　998 | 1,131　517 | 1,084　462 | 927　546 | 830　714 | 502　551 | 272　307 | 140　138 | 61　65 | 29　24 | −　　− | −　　− | −　　− | −　　− | −　　− |
| 2018 | 19,277 | −　　− | −　　− | 142 814 | 2,674 4,128 | 1,701　1,050 | 1,120　544 | 1,126　493 | 958　582 | 863　678 | 550　648 | 264　385 | 146　163 | 80　61 | 43　40 | −　　11 | −　　− | −　　− | −　　− | −　　− |
| 2019 | 19,774 | −　　− | −　　− | 123 809 | 2,800 4,091 | 1,706　1,084 | 1,151　579 | 1,111　496 | 952　578 | 865　708 | 629　726 | 338　373 | 166　185 | 97　89 | 45　34 | 19　15 | −　　− | −　　− | −　　− | −　　− |
| 2020 | 13,793 | −　　− | −　　− | 103 555 | 1,988 2,729 | 1,125　689 | 823　364 | 786　324 | 751　330 | 710　428 | 510　449 | 256　298 | 143　177 | 74　73 | 41　30 | 18　　− | −　　− | −　　− | −　　− | −　　− |
| 2021 | 15,878 | −　　− | −　　− | 153 646 | 2,474 3,291 | 1,411　830 | 958　414 | 803　335 | 690　371 | 689　456 | 580　486 | 333　346 | 156　177 | 87　71 | 50　37 | 12　14 | −　　− | −　　− | −　　− | −　　− |
