# Supplementary material for: Epidemiology of cruciate ligament surgery in Japan: A repeated cross-sectional study from 2014 to 2021
Source: PLoS One. 2023 Dec 22;18(12):e0288854. doi: 10.1371/journal.pone.0288854 (PMC10745212; doi:10.1371/journal.pone.0288854)
Supplement: S8 Table — To avoid the identification of individuals, aggregate units that are <10 in principle are not included. (DOCX) [file pone.0288854.s008.docx]

**S8 Table. Annual registrations of ligament reconstruction (K079) per 100,000 population according to age groups from 2014 to 2021.**

| Year | Total | Age groups (Upper: Male, Lower: Female) | | | | | | | | | | | | | | | | | | |
| --- | --- | --- | --- | --- | --- | --- | --- | --- | --- | --- | --- | --- | --- | --- | --- | --- | --- | --- | --- | --- |
|  |  | 0−4 | 5−9 | 10−14 | 15−19 | 20−24 | 25−29 | 30−34 | 35−39 | 40−44 | 45−49 | 50−54 | 55−59 | 60−64 | 65−69 | 70−74 | 75−79 | 80−84 | 85−89 | ≥90 |
| 2014 | 0.2 | −　　− | −　　− | −　　0.4 | 1.3　　1.7 | 0.8　　0.5 | 0.5　　− | 0.6 − | 0.4　　0.3 | 0.3　　− | 0.3　　− | −　　− | −　　− | −　　− | −　　− | −　　− | −　　− | −　　− | −　　− | −　　− |
| 2015 | 0.2 | −　　− | −　　− | −　　− | 1.2　　1.3 | 0.8　　0.3 | 0.7　　− | 0.5　　− | 0.4　　− | 0.2　　0.3 | −　　− | −　　− | −　　− | −　　− | −　　− | −　　− | −　　− | −　　− | −　　− | −　　− |
| 2016 | 0.2 | −　　− | −　　− | −　　− | 1.6　　1.0 | 0.6　　0.4 | 0.5　　− | 0.4　　− | 0.3　　− | 0.2　　− | 0.2　　− | −　　− | −　　− | −　　− | −　　− | −　　− | −　　− | −　　− | −　　− | −　　− |
| 2017 | 0.2 | −　　− | −　　− | −　　− | 0.8　　1.5 | 0.6　　0.4 | 0.4　　− | −　　− | 0.3　　0.3 | 0.2　　− | −　　− | −　　− | −　　− | −　　− | −　　− | −　　− | −　　− | −　　− | −　　− | −　　− |
| 2018 | 0.1 | −　　− | −　　− | −　　− | 0.6　　1.2 | 0.6　　− | −　　− | −　　− | −　　− | −　　− | −　　− | −　　− | −　　− | −　　− | −　　− | −　　− | −　　− | −　　− | −　　− | −　　− |
| 2019 | 0.1 | −　　− | −　　− | −　　− | 0.8　　0.9 | −　　− | 0.3 　− | 0.3　　0.3 | −　　− | −　　− | −　　− | −　　− | −　　− | −　　− | −　　− | −　　− | −　　− | −　　− | −　　− | −　　− |
| 2020 | 0.1 | −　　− | −　　− | −　　− | 0.5　　0.6 | −　　− | −　　− | −　　− | −　　− | −　　− | −　　− | −　　− | −　　− | −　　− | −　　− | −　　− | −　　− | −　　− | −　　− | −　　− |
| 2021 | 0.1 | −　　− | −　　− | −　　− | 0.4　　0.7 | −　　− | −　　− | −　　− | −　　− | −　　− | −　　− | −　　− | −　　− | −　　− | −　　− | −　　− | −　　− | −　　− | −　　− | −　　− |
