# Supplementary material for: Epidemiology of cruciate ligament surgery in Japan: A repeated cross-sectional study from 2014 to 2021
Source: PLoS One. 2023 Dec 22;18(12):e0288854. doi: 10.1371/journal.pone.0288854 (PMC10745212; doi:10.1371/journal.pone.0288854)
Supplement: S9 Table — To avoid the identification of individuals, aggregate units that are <10 in principle are not included. (DOCX) [file pone.0288854.s009.docx]

**S9 Table. Annual registrations of arthroscopic ligament reconstruction (K079-2) per 100,000 population according to age groups from 2014 to 2021.**

| Year | Total | Age groups (Upper: Male, Lower: Female) | | | | | | | | | | | | | | | | | | |
| --- | --- | --- | --- | --- | --- | --- | --- | --- | --- | --- | --- | --- | --- | --- | --- | --- | --- | --- | --- | --- |
|  |  | 0−4 | 5−9 | 10−14 | 15−19 | 20−24 | 25−29 | 30−34 | 35−39 | 40−44 | 45−49 | 50−54 | 55−59 | 60−64 | 65−69 | 70−74 | 75−79 | 80−84 | 85−89 | ≥90 |
| 2014 | 13.4 | −　　− | −　　− | 3.3 22.5 | 78.7 118.3 | 50.0 29.1 | 37.3　14.2 | 29.3 11.1 | 22.0 13.4 | 14.5 13.3 | 9.0 12.8 | 4.9　6.3 | 2.9 2.6 | 1.2 1.1 | 0.7 0.3 | −　　− | −　　− | −　　− | −　　− | −　　− |
| 2015 | 13.8 | −　　− | −　　− | 3.5 26.8 | 77.4 128.2 | 50.3　30.8 | 36.7　15.7 | 30.0 11.7 | 21.6 12.7 | 15.7 14.2 | 9.1 11.6 | 6.3 6.1 | 3.1 2.6 | 1.4 1.2 | 0.8 0.5 | −　　− | −　　− | −　　− | −　　− | −　　− |
| 2016 | 14.3 | −　　− | −　　− | 4.2 29.4 | 82.0 138.6 | 51.1　32.3 | 35.1　17.1 | 28.6 12.6 | 22.6 13.5 | 15.9 13.6 | 9.1 12.2 | 5.8　6.6 | 3.7　3.9 | 1.2 1.2 | 0.7 0.5 | −　　− | −　　− | −　　− | −　　− | −　　− |
| 2017 | 14.4 | −　　− | −　　− | 4.6 28.8 | 80.9 136.4 | 48.7　33.0 | 35.1　16.8 | 30.0 13.2 | 23.2 14.0 | 17.3 15.3 | 10.5 11.8 | 6.6　7.6 | 3.7　3.6 | 1.6 1.6 | 0.6 0.5 | −　　− | −　　− | −　　− | −　　− | −　　− |
| 2018 | 15.2 | −　　− | −　　− | 5.1 30.9 | 88.2 143.6 | 52.1　34.2 | 35.1　18.0 | 31.9 14.5 | 24.6 15.3 | 18.7 15.1 | 11.3 13.5 | 6.3　9.3 | 3.8 4.3 | 2.1 1.6 | 0.9 0.8 | −　　0.3 | −　　− | −　　− | −　　− | −　　− |
| 2019 | 15.7 | −　　− | −　　− | 4.5 31.0 | 93.8 144.3 | 51.7　35.1 | 35.8　19.1 | 32.2 15.0 | 24.9 15.5 | 19.6 16.5 | 12.7 15.0 | 7.8 8.8 | 4.3 4.8 | 2.6 2.3 | 1.1 0.8 | 0.5 0.3 | −　　− | −　　− | −　　− | −　　− |
| 2020 | 10.9 | −　　− | −　　− | 3.7. 21.2 | 67.9 98.2 | 34.8　22.3 | 25.1 11.7 | 22.9 9.9 | 19.7 8.9 | 16.5 10.2 | 10.2 9.2 | 5.8 6.9 | 3.6 4.5 | 2.0 1.9 | 1.0 0.7 | 0.4　　− | −　　− | −　　− | −　　− | −　　− |
| 2021 | 12.7 | −　　− | −　　− | 5.6 24.7 | 86.4 121.2 | 44.0　27.1 | 29.2 13.3 | 24.0 10.5 | 18.5 10.3 | 16.6 11.3 | 11.8 10.1 | 7.1 7.5 | 4.0 4.5 | 2.4 1.9 | 1.3 0.9 | 0.3 0.3 | −　　− | −　　− | −　　− | −　　− |
